# Cinnamon Essential Oil: A Promising Natural Strategy for the Prevention of Rabbit Colibacillosis

**DOI:** 10.3390/antibiotics15090840

**Published:** 2026-08-30

**Authors:** Gaia Casalino, Davide Messina, Roberta Tardugno, Giuseppe Fracchiolla, Giancarlo Bozzo, Dalila Salierno, Francesco D’Amico, Martina Canale, Antonella Bove, Antonio Camarda, Elena Circella

**Affiliations:** 1 Department of Veterinary Medicine, University of Bari, S. P. Casamassima km 3, 70010 Valenzano, Italy; giancarlo.bozzo@uniba.it (G.B.); d.salierno1@phd.uniba.it (D.S.); m.canale1@studenti.uniba.it (M.C.); antonella.bove@uniba.it (A.B.); antonio.camarda@uniba.it (A.C.); elena.circella@uniba.it (E.C.); 2 Division of Veterinary Clinical Science, School of Veterinary Medicine and Science, Faculty of Medicine and Health Sciences, University of Nottingham, Sutton Bonington Campus, Loughborough LE12 5RD, UK; davide.messina1@nottingham.ac.uk; 3 Department of Pharmacy-Drug Sciences, University of Bari Aldo Moro, Via Orabona, 4, 70125 Bari, Italy; roberta.tardugno@uniba.it (R.T.); giuseppe.fracchiolla@uniba.it (G.F.); 4 Dipartimento della Prevenzione-S.C. Sanità Animale, Azienda Sanitaria Locale di Alessandria, distretto di Tortona Corso Romita 25-B, 15057 Tortona, Italy; fdamico@aslal.it

**Keywords:** colibacillosis, *Escherichia coli*, rabbit, enteritis, cinnamon, cinnamaldehyde

## Abstract

Background/Objectives: Colibacillosis, caused by enteropathogenic strains of *Escherichia coli* (EPEC), is one of the most common infectious diseases in rabbit farms. In the past, antimicrobials were widely used to control colibacillosis, but their use in prevention is currently prohibited. Therefore, the adoption of new prevention strategies as alternatives to the use of antibiotics is very important. The aim of this study was to evaluate the antimicrobial efficacy of cinnamon essential oil (*Cinnamomum zeylanicum*) against EPEC strains responsible for colibacillosis in rabbits. Methods: For the experiment, 124 strains of *E. coli* isolated from rabbits that had died of colibacillosis in 10 industrial farms were used. Suspensions of each strain prepared according to CLSI standards with a bacterial density of 1 × 10^8^ CFU/mL were tested using 100% pure commercial cinnamon essential oil (CEO) at concentrations ranging from 0.2 to 0.8 μL/mL. Minimum inhibitory concentrations MIC_50_ and MIC_90_ were determined according to a protocol described previously. Results: MIC_50_ and MIC_90_ were 0.5 μL/mL and 0.6 μL/mL, inhibiting 64.5% and 94.3% of strains, respectively. Furthermore, strains exposed to 0.4 μL/mL were 12.9 times more likely to be inhibited than those exposed to 0.3 μL/mL (*p* = 0.0001). Conclusions: CEO was found to be effective against *E. coli* strains tested at high bacterial densities that can usually be found per gram of faeces in rabbits affected by colibacillosis. Consequently, cinnamon should be useful in preventing colibacillosis by limiting the replication of *E. coli*, as it is usually found in lower densities in the intestines of healthy rabbits, preventing the onset of colibacillosis and reducing the use of antibiotics in rabbit farms.

## 1. Introduction

Although rabbits are now widely kept as pets, Spain, France, and Italy continue to be major producers of rabbit meat in Europe [1]. Based on European recommendations on the welfare of rabbits in meat production systems, the shift from conventional cage systems to alternative systems such as enriched cages, outdoor areas with full or partial access, ground pens with raised platforms, and the adoption of less intensive cycles is encouraged. These measures aim to improve both the productive performance and health conditions of rabbits, leading to a decrease in mortality rates throughout the entire reproductive cycle and, consequently, in the use of antibiotics. Improving the welfare conditions of rabbits is essential to prevent the development of diseases, particularly those of multifactorial aetiology that represent the health problems most frequently observed in rabbits [2].

Amongst the conditioned diseases, digestive disorders are responsible for mortality and symptoms that range from temporary decrease in feed intake, weight loss and mild diarrhoea in subclinical forms, to total refusal to eat, acute diarrhoea and/or caecal occlusion in acute forms. Rabbits in the weaning and post-weaning period, from the third to fourth week of age and beyond, are particularly susceptible to enteric syndromes caused mainly by bacteria and protozoa [3,4,5]. Among bacteria, *Escherichia* (*E*.) *coli* is one of the most involved [3,4,5]. *Enteropathogenic Escherichia coli* (*EPEC*) is considered the main pathotype responsible for colibacillosis in rabbits. This disease is associated with several predisposing factors such as poor environmental control, poor hygiene conditions, crowding and stress, inadequate nutrition, sudden changes in diet, genetics, and other concomitant diseases that can promote the massive replication of *E. coli* in the intestine of rabbits [6]. EPEC strains belong to the group known as attaching–effacing Escherichia coli (AEEC) [7], whose pathogenic mechanism is based on adhesion and the subsequent destruction of intestinal cells [8,9,10]. Considering that colibacillosis is one of the most frequent pathologies in industrial rabbit farms in most rabbit meat-producing countries of Europe, such as Spain, France, Italy and Portugal [5,11,12,13], the control of EPEC strains is a critical issue in rabbit farming. In the past, antimicrobials were used extensively in rabbit farms to prevent colibacillosis. However, the misuse and abuse of antimicrobials are among the main factors contributing to the emergence of antimicrobial resistance (AMR). *E. coli* is one of the bacteria most frequently involved in AMR. According to the 2022 report of the Global Antimicrobial Resistance and Use Surveillance System (GLASS), *E. coli* strains showed a 42% resistance rate to third-generation cephalosporins. Therefore, Regulation (EU) 2019/6 and Circular No. 1/2022 on guidelines for the prudent use of antibiotics in rabbit farming for meat production have led to a ban on the preventive use of antibiotics in European Union countries increasing the interest on different strategies of prevention. Since colibacillosis is a conditioned disease of rabbits, it is possible to implement a control strategy based on improving farming conditions and other methods such as alternative preventive measures to antibiotics. The potential antimicrobial activity of certain natural substances could represent one such control strategy [14]. Therefore, the aim of this study was to evaluate the antimicrobial efficacy of cinnamon (*Cinnamomum zeylanicum*) essential oil against strains of enteropathogenic *E. coli* (EPEC) isolated from rabbits that died of colibacillosis in industrial farms.

## 2. Materials and Methods

### 2.1. Cinnamon Essential Oil (CEO) and Its Phytochemical Composition by Gas Chromatography-Mass Spectrometry (GC-MS) (Waldbronn, Germany)

A commercial, 100% pure essential oil obtained from the bark of cinnamon (*Cinnamomum zeylanicum*) through hydro-distillation was used in the experiment.

The GC-MS analysis was conducted according to the reported methodology [15]. *C. zeylanicum* bark essential oil solution 1:100 *v*/*v* in ethyl acetate was analysed by the GC-MS technique using an Agilent 6890N GC coupled with a 5973N MSD HP ChemStation (Waldbronn, Germany). Compounds were separated on an Agilent Technologies HP-5 MS cross-linked poly-5% diphenyl–95% dimethyl polysiloxane capillary column (Waldbronn, Germany). The column temperature was initially set at 40 °C for 5 min, then increased at a rate of 4 °C/min up to 280 °C. MS detection was performed with electron ionisation (EI) at 70 eV, operating in the full-scan acquisition mode in the *m*/*z* range 40–400. Compounds were identified by comparing the retention times of the chromatographic peaks with those of authentic reference standards run under the same conditions and by comparing the LRIs relative to C8-C20 *n*-alkanes obtained on the HP-5 column under the same mentioned conditions with the literature [16]. Comparison of the MS-fragmentation pattern of the target analytes with those of pure components was performed. A mass-spectrum database search was carried out by using the National Institute of Standards and Technology (NIST, Gaithersburg, MD, USA) mass spectral database (version NIST 17, 2017). Semi-quantification was calculated as the relative percentage amount of each analyte (% Area); in particular the values were expressed as the percentage peak area relative to the total composition of *C. zeylanicum* bark essential oil.

### 2.2. Bacterial Strains

The study used 124 strains of *E. coli* collected between 2001 and 2024 from 10 industrial rabbit farms for meat production. All *E. coli* strains were isolated from rabbits that had died from colibacillosis. Samples of intestinal contents from each rabbit were plated on MacConkey agar (Oxoid, Basingstoke, UK), which was incubated at 37 °C for 24 h under aerobic conditions. Each colony morphologically compatible with *E. coli* was transferred to tryptic soy agar (TSA) (Oxoid, Basingstoke, UK), incubated under the same conditions, and identified using API-20E (bioMérieux, Marcy l’Étoile, Lyon, France). The isolates were characterised as enteropathogenic *E. coli* (EPEC) following PCR detection of the *eae* and *afr2* genes, as previously described [17,18], before being included in the strain collection. Each *E. coli* strain was stored in Brucella broth with glycerol (10%) at −20 °C at the Avian Diseases Section of the Department of Veterinary Medicine in Bari.

### 2.3. Preparation of Bacterial Suspensions and Culture Medium for the Efficacy Tests

A bacterial density of 1 × 10^8^ CFU/mL was chosen to evaluate the efficacy of the CEO. All strains were cultured on TSA at 37 °C overnight prior to testing. Bacterial suspensions were prepared from each strain by dissolving the colonies with a sterile loop in 5 mL of sterile saline solution (0.9%) until reaching 0.5 on the McFarland standard, corresponding to 1 × 10^8^ CFU/mL, according to the CLSI guidelines [19].

Efficacy tests were performed using Mueller–Hinton agar (Oxoid), reconstituted in distilled water according to the manufacturer’s instructions and autoclaved at 121 °C for 15 min. The medium was then heated to 50 °C in a thermostatic bath before adding CEO, previously diluted in DMSO in the ratio of 9:1 (9 parts CEO to one-part DMSO) according to [20], in different concentrations, chosen based on the results of our previous studies [21]. The addition of the essential oil was carried out when the agar was fluid since it was brought to 50 °C in the thermostatic bath. The agar was then stirred for 30 s using an automatic stirrer to achieve the best distribution of the essential oil in the fluid and then plated. CEO concentrations ranging from 0.02% to 0.08% (0.02, 0.03, 0.04, 0.05, 0.06, 0.07, 0.08%) and corresponding to 0.2–0.8 µL/mL (0.2, 0.3, 0.4, 0.5, 0.6, 0.7, 0.8 µL/mL) were used.

### 2.4. Efficacy Testing and Evaluation of MIC_50_ and MIC_90_ Values

**Figure 1:**
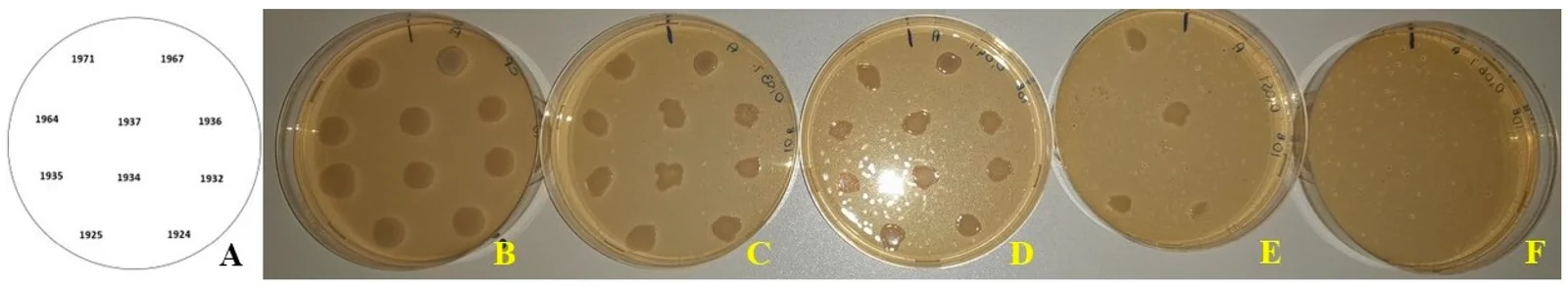
Efficacy of CEO on *E. coli* strains from rabbits (*spot* inoculation). (**A**): one of the numbered diagrams for strain identification. (**B**): positive control for growth of strains. (**C**–**F**): inhibitory effect based on the concentrations of CEO used; (**C**) (0.03%) and (**D**) (0.04%): slight inhibitory effect evidenced by the gradual reduction in the size of the bacterial drop; (**E**): (0.05%): complete inhibitory effect on 6 strains that are no longer visible; (**F**) (0.06%): complete inhibitory effect on all 10 strains.

Based on previous studies [21,22], 10 µL of each bacterial suspension was drop-deposited, well-spaced as shown in Figure 1, onto Mueller–Hinton agar containing CEO concentrations as reported above. A series of numbered diagrams was prepared to identify the different strains of *E. coli* both during test preparation and when reading the results.

Using these diagrams, ten different strains were analysed simultaneously on each plate. Each strain was also inoculated on Mueller–Hinton agar without CEO containing DMSO at the highest concentration used in the trials to obtain a positive control for bacterial growth. All plates were incubated at 37 °C overnight. CEO inhibitory efficacy was based on growth/no growth at the point of inoculation. In particular, as already done for the test of a previous study [22], the results were read by two operators using a light and placing a dark screen under the plates. The effectiveness of the CEO was only taken into account in the event of a complete absence of visible colonies. Partial growth or the presence of a single colony was considered evidence of ineffectiveness. In these cases, reading was supported by taking photographs so that the enlarged image could be controlled. According to [21], the minimum inhibitory concentration (MIC) was defined as the minimum concentration of CEO at which no growth was observed at the inoculation site. Each experiment was repeated twice on two different days. Therefore, MIC_50_ and MIC_90_ values were calculated as the minimum concentrations of CEO capable of inhibiting 50% and 90% of the bacterial strains tested, respectively.

### 2.5. Statistical Analysis

Statistical analysis was performed using Microsoft^®^ Excel^®^ for Microsoft 365 MSO (Version 2510 Build 16.0.19328.20244) 64-bit and MedCalc Software Odds ratio calculator version 23.4.2.

## 3. Results

### 3.1. GC-MS Phytochemical Composition of CEO

The *Cinnamomum zeylanicum* bark essential oil was characterised via GC-MS analysis, resulting in the identification of 24 constituents representing 98.13% of the total composition (Table 1). Phenylpropanoids and aromatic compounds constituted the most abundant chemical class (62.73%), with *(Z)*-cinnamaldehyde being the primary constituent (48.93%), followed by eugenol (11.86%). The chemical profile is consistent with the *C. zeylanicum* bark essential oil profile reported in the literature, with some quantitative differences [23,24]. Specifically, the relative abundance of the main aldehyde was lower in the analysed sample 49% than the reported 68% [23,24], while eugenol (12%) and beta-caryophyllene (15%) contents were higher than the values reported. These quantitative fluctuations in the ratios of cinnamaldehyde, eugenol, and β-caryophyllene, as well as in other minor metabolites, could be attributable to variations in agro-climatic factors, geographical origin, or hydrodistillation parameters, which are documented factors influencing the phytochemical composition of essential oils.

### 3.2. Efficacy Testing and Evaluation of MIC_50_ and MIC_90_ Values

MIC_50_ was found to be 0.5 µL/mL, with the inhibition of 80 out of 124 strains analysed (64.5%), while the MIC_90_ value was 0.6 µL/mL, with inhibition of 117 strains (94.3%) (Table 2). Seven strains (5.6%) were inhibited at 0.3 µL/mL of CEO.

The MIC values obtained for the strains, based on their farm of origin, are shown in Table 3.

In most cases, as for the strains isolated from rabbits on farms 1, 2, 3, 5, 6, 7 and 9, the MIC_90_ value was 0.6 µL/mL. However, for strains from farms 4 and 10, it was 0.4 µL/mL, and for those isolated from rabbits on farm 8, it was 0.7 µL/mL. The MIC_50_ was 0.4 (strains from farms 4, 7, 9, and 10) or 0.5 µL/mL (strains from farms 1, 2, 5, and 6). In contrast, it was 0.6 µL/mL for strains from farm 8. In farms 1, 4 and 9, some strains were inhibited by 0.3 µL/mL of CEO.

Overall, there was a positive trend, as indicated by the fact that the percentage of inhibited *E. coli* strains increased with the CEO concentrations used.

Regarding the differences in inhibited *E. coli* strains between different CEO concentrations, 37.9% (95% CI: 27.8387% to 47.1475%; *p* < 0.0001) more *E. coli* strains were inhibited at a concentration of 0.4 μL/mL as opposed to 0.3 μL/mL, whilst 29.8% (95% CI: 20.1609% to 39.0112%; *p* < 0.0001) and 5.7% (95% CI: 1.5223% to 11.2648%; *p* = 0.0071) more *E. coli* strains were inhibited at a concentration of 0.6 μL/mL as opposed to 0.5 μL/mL and at a concentration of 0.7 μL/mL as opposed to 0.6 μL/mL, respectively (Table 3). Regarding the association between cinnamon concentrations and inhibition, *E. coli* strains that were exposed to a 0.4 μL/mL CEO concentration had an OR 12.8939 (95% CI: 5.5596 to 29.9035; *p* = 0.0001), meaning that they were 12.8939 times more likely to be inhibited than those exposed to 0.3 μL/mL CEO concentration, whilst those that were exposed to 0.6 μL/mL and 0.7 μL/mL concentrations had an OR 9.1929 (95% CI: 3.9420 to 21.4380; *p* < 0.0001) and 15.8936 (95% CI: 0.8977 to 281.3949; *p* = 0.0592), respectively (Table 4).

## 4. Discussion

Based on the data obtained, cinnamon essential oil appears to have antimicrobial activity against *E. coli* strains of rabbits. The results are likely attributable to cinnamaldehyde, the main active component found in the cinnamon essential oil tested in this study, which is effective against bacteria belonging to different species [25], in synergy with benzyl alcohol and eugenol, which are other compounds present in the essential oil. Cinnamaldehyde appears to be able to penetrate the phospholipid bilayer of bacterial cell walls, interfering with the cell in various ways. Generally, the outer lipopolysaccharide membrane of Gram-negative bacteria limits the penetration of lipophilic compounds, making these substances potentially more active against Gram-positive bacteria [26,27]. Interestingly, the high efficacy observed in this study against EPEC strains, which have a thick outer layer of lipopolysaccharides, may have been linked to and enhanced by other mechanisms of action of the amount of cinnamaldehyde that manages to penetrate the bacterial cell. In fact, cinnamaldehyde can cause cytoplasmic coagulation, denaturation of enzymes and proteins and loss of metabolites and ions [28,29]. In addition, cinnamaldehyde hinders biofilm formation by interfering with bacterial proliferation [30]. One of the most interesting properties of cinnamaldehyde is the anti-adhesive mechanism [31], which inhibits specific adhesins, preventing the bacterium from adhering to tissues, making it particularly effective against *E. coli* strains from rabbits, as they belong to the EPEC pathotype. The efficacy against EPEC strains observed in this study appears particularly promising, especially considering the bacterial density (10^8^ CFU/mL) that we chose to use for the experiments, that is the amount of *E. coli* that can be found per gram of faeces in rabbits affected by colibacillosis [32]. Of course, the use of cinnamon for therapeutic purposes is currently unthinkable, and antibiotics remain the only method of treating colibacillosis. However, based on the results of this study, cinnamon could be used successfully to prevent colibacillosis, as a lower *E. coli* density, between 10^3^ and 10^5^ CFU/g of faeces, is usually found in healthy individuals aged between 42 and 49 days, which is the age at which rabbits are most susceptible to colibacillosis [33].

Therefore, the use of cinnamon could be useful in preventing clinical forms by limiting *E. coli* replication in the rabbit intestine, along with the control of environmental, management, and nutritional factors that, under farm conditions, can promote *E. coli* proliferation and predispose rabbits to develop the colibacillosis [2,3,5,34,35]. If confirmed under field conditions, this preventive strategy could help reduce the occurrence of colibacillosis resulting in reduced antimicrobial treatments in rabbit farms, thus contributing to the responsible use of antimicrobials and reducing the selective pressure associated with the emergence of antimicrobial-resistant bacteria. Some slight differences in efficacy based on the *E. coli* strains tested were found. These results suggest the utility of testing the specific susceptibility of EPEC strains spread among the rabbits of a farm, on a case-by-case basis, to the essential oil before its possible use in the field, similar to performing an antibiogram or MIC test that usually precedes the choice and use of an antibiotic [36,37,38,39]. In the case of natural substances, unlike antibiotic susceptibility testing, preliminary tests will be aimed at assessing the quantities of essential oil to be used rather than assessing any resistance. Indeed, natural compounds base their antimicrobial activity on different mechanisms involving different parts of the bacterial cell [40,41], making it more difficult for bacteria to develop resistance mechanisms [42,43]. Susceptibility testing to natural compounds could be useful to evaluate the specific amount of compound that should be most effective in the specific situation because small differences in strain susceptibility could lead to large differences in dosage to be used in farms where there are a high number of rabbits to be treated. Given that treatments in rabbit farms are usually administered via drinking water or feed [44], differences of 0.1–0.2 microliters per mL in vitro become 10–20 mL per quintal of feed water in field. In addition, to be effective, natural compounds must be administered daily for a long time. These factors may make the use of essential oil not cost-effective on some farms because it may become too expensive.

## 5. Conclusions

In conclusion, this study highlights the interesting in vitro antimicrobial efficacy of cinnamon essential oil against enteropathogenic strains of *E. coli*.

This finding suggests the possible use of cinnamon essential oil as a supportive strategy in the prevention of colibacillosis in rabbit farms, although further investigation under field conditions is needed. The use of cinnamon essential oil in association with improved management and hygiene conditions of farms could lead to a decrease in colibacillosis in rabbits and, consequently, a reduction in the use of antibiotics on farms. Such an approach could support more sustainable rabbit production and align with One Health strategies aimed at limiting antimicrobial resistance while protecting animals, environmental, and public health. However, further in vivo studies are needed before practical application can be recommended.

Moreover, further research is needed to evaluate the most appropriate method of administration, the palatability of food or water supplemented with essential oils, and the possible effects of essential oils on the entire intestinal microbiota of rabbits.

## Figures and Tables

**Table 1:** *Cinnamomum zeylanicum* bark essential oil GC-MS phytochemical composition.

Compound ^a^	LRI Exp ^b^	LRI Lit ^c^	% Area
α-pinene	937	939	3.18
camphene	948	954	1.13
benzaldehyde	963	960	0.49
β-pinene	978	979	1.08
α-phellandrene	1006	1002	2.25
Δ3-carene	1007	1011	0.23
α-terpinene	1016	1017	0.24
*p*-cymene	1022	1024	3.66
limonene	1025	1029	1.87
1,8-cineole	1039	1031	0.50
α-terpinolene	1087	1088	0.15
linalool	1104	1096	4.60
terpinen-4-ol	1173	1177	0.11
α-terpineol	1187	1188	0.23
(Z)-cinnamaldehyde	1263	1219	48.93
safrole	1285	1287	0.69
eugenol	1363	1359	11.86
α-copaene	1382	1376	0.44
β-caryophyllene	1428	1419	14.93
(Z)-cinnamyl acetate	1439	1389	0.18
humulene	1460	1454	0.33
eugenyl acetate	1528	1524	0.33
caryophyllene oxide	1589	1583	0.47
benzyl benzoate	1766	1760	0.25
Monoterpene hydrocarbons			13.79
Oxygenated monoterpenes			5.44
Phenylpropanoids and aromatic compounds			62.73
Sesquiterpene hydrocarbons			15.70
Oxygenated sesquiterpenes			0.47
Total identified			98.13

^a^ Compounds are listed in elution order from the HP-5MS silica capillary column. ^b^ Experimental Linear Retention Indices (LRIexp) relative to *n*-alkanes (C8–C20) on the same HP-5MS column. ^c^ Literature Linear Retention Indices (LRIlit) from R.P. Adams 2007 [16].

**Table 2:** Inhibitory effect of cinnamon essential oil against *E. coli* strains from rabbits.

Cinnamon Essential Oil (µL/mL)	N° Inhibited Strains/ No. of Analysed Strains	% of Inhibition
0.2	0/124	0
0.3	7/124	5.6
0.4	54/124	43.5
0.5	80/124	64.5
0.6	117/124	94.3
0.7	124/124	100
0.8	124/124	100

**Table 3:** Inhibitory efficacy of CEO against *E. coli* strains, divided according to farm of origin.

		Cinnamon Concentrations (μL/mL) No Inhibited Strains (%)				
Farm	N° of Strains	0.3 µL/mL	0.4 µL/mL	0.5 µL/mL	0.6 µL/mL	0.7 µL/mL
1	24	1 (4.2)	9 (37.5)	19 (79.2)	23 (95.8)	24 (100)
2	22	0 (0)	10 (45.4)	11 (50)	21 (95.4)	22 (100)
3	17	0 (0)	1 (5.9)	4 (23.5)	16 (94.1)	17 (100)
4	16	5 (31.2)	16 (100)	16 (100)	16 (100)	16 (100)
5	11	0 (0)	4 (36.4)	7 (63.6)	10 (90.9)	11 (100)
6	10	0 (0)	2 (20)	6 (60)	10 (100)	10 (100)
7	8	0 (0)	4 (50)	7 (87.5)	8 (100)	8 (100)
8	6	0 (0)	0 (0)	1 (16.7)	3 (50)	6 (100)
9	6	1 (16.7)	4 (66.7)	5 (83.3)	6 (100)	6 (100)
10	4	0 (0)	4 (100)	4 (100)	4 (100)	4 (100)

**Table 4:** Differences in the percentage of *E. coli*-inhibited strains between different CEO concentrations and the association between concentrations and strains inhibited.

	Differences in % of *E. coli* Inhibited Strains Between Different CEO Concentrations			Association Between CEO Concentrations and *E. coli* Strains Inhibited		
Cinnamon Essential Oil Concentrations (μL/mL)	Difference	95% CI	*p*-Value	OR	95% CI	*p*-Value
0.3 and 0.4	37.9%	27.8387% to 47.1475%	<0.0001	12.8939	5.5596 to 29.9035	0.0001
0.3 and 0.5	58.9%	48.5520% to 67.2771%	<0.0001	30.3896	13.0314 to 70.8693	0.0001
0.3 and 0.6	88.7%	80.8498% to 92.7810%	<0.0001	279.3673	95.0096 to 821.4547	0.0001
0.3 and 0.7	94.4%	88.1001% to 97.2688%	<0.0001	3901.0000	220.3345 to 69,066.8079	0.0001
0.4 and 0.5	21.0%	8.6021% to 32.5116%	0.0009	2.3569	1.4136 to 3.9297	0.0010
0.4 and 0.6	50.8%	40.3955% to 59.6879%	<0.0001	21.6667	9.3423 to 50.2493	<0.0001
0.4 and 0.7	56.5%	47.2094% to 64.9006%	<0.0001	322.1009	19.5904 to 5295.9232	0.0001
0.5 and 0.6	29.8%	20.1609% to 39.0112%	<0.0001	9.1929	3.9420 to 21.4380	<0.0001
0.5 and 0.7	35.5%	27.0754% to 44.2419%	<0.0001	137.6460	8.3582 to 2266.8173	0.0006
0.6 and 0.7	5.7%	1.5223% to 11.2648%	0.0071	15.8936	0.8977 to 281.3949	0.0592 *

* Non-Statistically significant results.

## Data Availability

Data is contained within the article.
